# Ecology of Subglacial Lake Vostok (Antarctica), Based on Metagenomic/Metatranscriptomic Analyses of Accretion Ice

**DOI:** 10.3390/biology2020629

**Published:** 2013-03-28

**Authors:** Scott O. Rogers, Yury M. Shtarkman, Zeynep A. Koçer, Robyn Edgar, Ram Veerapaneni, Tom D’Elia

**Affiliations:** 1 Department of Biological Sciences, Bowling Green State University, Bowling Green, OH 43403, USA; E-Mails: yshtark@bgsu.edu (Y.M.S.); zeynep.kocer@stjude.org (Z.A.K.); redgar@falcon.bgsu.edu (R.E.); 2 Department of Biological Sciences, Bowling Green State University, Firelands Campus, Huron, OH 44839, USA; E-Mail: ramv@bgsu.edu; 3 Biological Sciences, Indian River State College, 32021 Virginia Avenue, Fort Pierce, FL 34981, USA; E-Mail: tdelia@irsc.edu

**Keywords:** Lake Vostok, subglacial lake, metagenomic, metatranscriptomic, marine, aquatic

## Abstract

Lake Vostok is the largest of the nearly 400 subglacial Antarctic lakes and has been continuously buried by glacial ice for 15 million years. Extreme cold, heat (from possible hydrothermal activity), pressure (from the overriding glacier) and dissolved oxygen (delivered by melting meteoric ice), in addition to limited nutrients and complete darkness, combine to produce one of the most extreme environments on Earth. Metagenomic/metatranscriptomic analyses of ice that accreted over a shallow embayment and over the southern main lake basin indicate the presence of thousands of species of organisms (94% Bacteria, 6% Eukarya, and two Archaea). The predominant bacterial sequences were closest to those from species of Firmicutes, Proteobacteria and Actinobacteria, while the predominant eukaryotic sequences were most similar to those from species of ascomycetous and basidiomycetous Fungi. Based on the sequence data, the lake appears to contain a mixture of autotrophs and heterotrophs capable of performing nitrogen fixation, nitrogen cycling, carbon fixation and nutrient recycling. Sequences closest to those of psychrophiles and thermophiles indicate a cold lake with possible hydrothermal activity. Sequences most similar to those from marine and aquatic species suggest the presence of marine and freshwater regions.

## 1. Introduction

**Figure 1:**
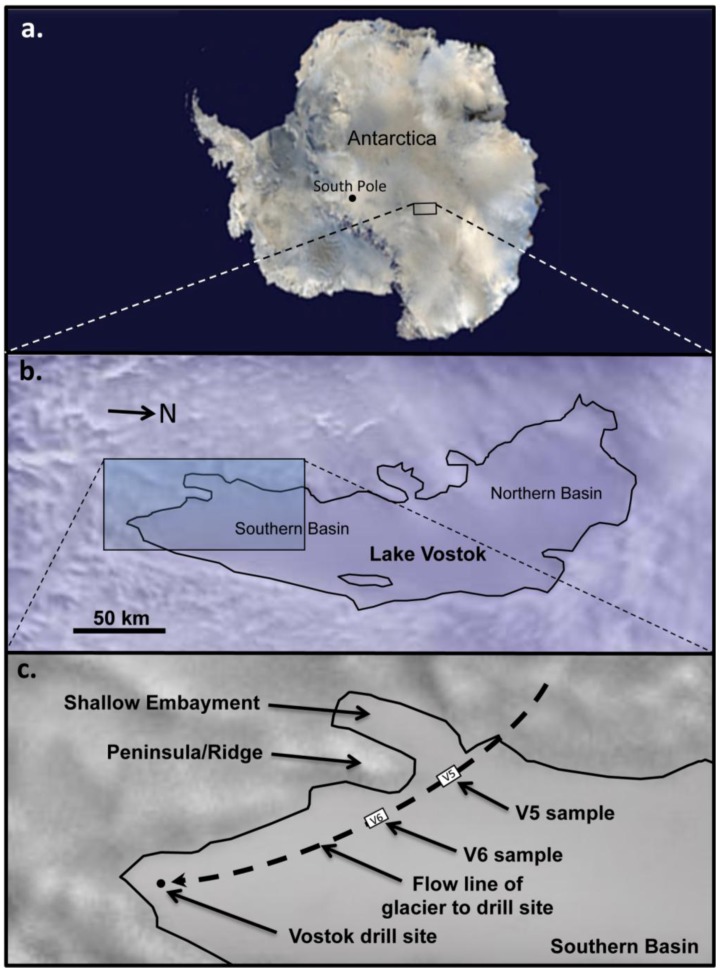
Source of ice core sections used in this study. (**a**) Location of Lake Vostok (small rectangle) in Antarctica; (**b**) Detail of the outline of Lake Vostok, as indicated by radar [1,8,23,24]; (**c**) Detail of the southern end of Lake Vostok, showing the locations of the shallow embayment, ridge, southern basin, track of glacier to the drill site (dashed line), and approximate locations where the accretion ice samples (V5 and V6) were formed.

Nearly 400 subglacial lakes have been discovered in Antarctica, the largest of which is Lake Vostok [1,2,3,4,5]. While Lake Vostok covers an area (15,690 km^2^) that is about 80% of the size of the Laurentian Great Lake Ontario, it holds a larger volume of water (5,400 km^3^), due to its depth (maximum depth = 510 m). It lies beneath 3,700 to 4,200 m of ice, and has been continuously ice-covered for the past 15 million years, with the only known influx of water originating from melting of the overriding glacier. Lake Vostok consists of a northern and the southern basin (Figure 1). Little is known about the northern basin, but more is known about the southern basin because of studies based on an ice core that was drilled over the southeastern corner of the lake. While glacial ice melts over portions of the lake, water from the lake freezes (*i.e.*, accretes) to the bottom of the glacier in other parts of the lake creating an accretion ice layer that is over 200 m thick in some locales [2,6,7,8]. Because the glacier moves across the lake at a rate of approximately 3 m per year, the accretion ice holds a temporal record that spans approximately 5,000 to 20,000 years [7], as well as a spatial record of the surface waters of the lake. The accretion ice from the core represents several parts of the southern portion of the lake, including a region near a shallow embayment on the southwestern corner of the lake and the southern portions of the southern main basin.

Accretion ice from the ice core has been analyzed to determine the concentrations of specific ions (e.g., Na^+^, K^+^, Ca^2+^, Cl^−^, SO_4_^2−^) [9,10,11,12] and biomass [13,14,15,16,17,18,19] originating from several locations in the lake. The ice that formed in the vicinity of the shallow embayment (3,538–3,608 m, termed type 1 accretion ice) contains fine particulate matter [7], as well as relatively high concentrations of ions [9], organisms and nucleic acids [9,16,17]. Conversely, accretion ice that formed over the southern basin (3,609–3,769 m, termed type 2 ice) contains low concentrations of particulates, ions, organisms and nucleic acids. Dozens of bacterial cells, fungal cells and sequences have been reported from several accretion ice core sections [12,13,14,15,16,17,18,19] with the highest numbers concentrated in the core sections that represent regions of the lake near the shallow embayment. They include many types of organisms that are common to other aquatic environments, as well as many that remain unidentified. Sequences from thermophilic bacteria have been reported, indicative of possible hydrothermal activity in the lake [20,21,22]. Autotrophic and heterotrophic species have also been reported from the accretion ice [16,17]. These reports indicate that Lake Vostok might be more biologically complex than previously concluded.

Another feature of Lake Vostok is that it lies entirely below current mean sea level. Its surface is more than 200 m below sea level, and the deepest point is almost 800 m below sea level. A recent study [23] concluded that Lake Vostok lies within a graben (similar to those in the Great Rift Valley in Africa) that formed more than 60 million years ago. A second study, based on radar data [24], reported that 35 million years ago when Antarctica was free of ice, the Southern Ocean was in the immediate vicinity of Lake Vostok. However, by 34 million years ago, ice had covered the lake and lowered sea level, which might have isolated it from a direct connection to the ocean. The fact that parts of Lake Vostok contain moderate levels of salt, and that sequences from marine organisms have been detected in the accretion ice indicate that this lake might have a complex history. In this study, we utilize metagenomic/metatranscriptomic sequence data ([22]; Supplementary Tables S1–S10) to reconstruct the possible ecology of Lake Vostok.

**Table 1:** Summary of sequence results for V5 (total of 1,863; or 3,718, including sequences that cannot be classified to species). The number of sequences in each taxon, ecology/physiology of each classified taxon and species characteristics are presented. Sequences are grouped by Domain and Phylum.

Taxon	Unique gene sequences	Unique rRNA gene sequences ^a^		Ecology and physiology ^b^	Species characteristics ^b^
		≥200 nt <200 nt			
**BACTERIA**	**3495**	**2535**	**460**		
**Acidobacteria**	2	1	0	acidophilic, soil, adaptable	chemoorganotrophic heterotrophs
**Actinobacteria**	228	151	24	thermophilic, halotolerant, psychrotolerant, alkalaitolerant, psychrophilic, Antarctic, deep sea sediments, lake sediments, some grow on limestone	nitrogen fixation, nitrite oxidation, ammonia oxidation, organic decomposition, heterotrophs
**Bacterioidetes/Chlorobi**	88	61	8	aquatic, sediments, thermophilic, psychrophilic, alkalaiphilic, anaerobic	carbon fixation (use sulfide ions, hydrogen or ferrous ions), reductive TCA cycle
**Chloroflexi**	1	0	0	aerobic, thermophilic	carbon fixation using the 3-hydroxylpropionic bicycle
**Cyanobacteria**	228	144	60	common in Antarctic lakes, at least one is thermophilic (*Thermosynecoccus* sp.)	carbon fixation using the reductive pentose phosphate cycle, some are from anoxygenic ancestors
**Deferribacteres**	1	1	0	animal intestines, anaerobic	chemoorganotrophic heterotrophic
**Deinococcus/ Thermus**	5	1	1	thermophilic, radiophilic, aerobic some associate with cyanobacteria	chemoorganotrophic heterotrophic
**Fibrobacteres**	1	0	1	anaerobic, inhabit animal intestines	chemoorganotrophic heterotrophic
**Firmicutes**	602	401	40	Spore formers, common in extreme environments, thermophiles, mesophilic, psychrophilic, psychrotolerant, halophilic, hot springs, deep sea thermophilic, anaerobic, aerobic	heterotrophic
**Fusobacteria**	10	8	0	parasitic on animals, anaerobic	chemoorganotrophic heterotrophic
**Planctomycetes**	6	2	2	Fresh, brackish and saline lakes/ponds, anaerobic	chemoautolithotrophic anammox, nitrite reduction using ammonium as electron donor
**Proteobacteria**	474	265	46		
Alphaproteobacteria	91	45	7	Psychrophilic, mesophilic, thermophilic, Antarctic lakes, animal symbionts, aerobic, soil/sediments, aquatic, alkalaitolerant, require calcium, marine, halotolerant	nitrite reduction, nitrifying bacteria, denitrification (nitrate to nitrogen gas), methylotrophic, use inorganic sulfur, oxidize sulfate and thiosulfate, carbon fixation using the reductive pentose phosphate cycle, carbon fixation using the reductive TCA cycle
Betaproteobacteria	105	31	7	thermophilic, mesophilic, psychrophilic, aquatic, aerobic, highly adaptable	nitrogen fixation, nitrate reduction, ammonia oxidation, carbon fixation using the reductive pentose phosphate cycle, manganese oxidation, iron oxidation, inorganic sulfur oxidation, arsenic oxidation
Deltaproteobacteria	10	5	0	aquatic, soil, mesophilic, anaerobic, aerobic, freshwater debris, predator of Gram-negative bacteria, halotolerant, marine	carbon fixation using the reductive TCA cycle, iron reduction, sulfur reduction, ethanol fermentation
Epsilonproteobacteria	6	3	2	Some animal associated, mesophilic, thermophilic, aerobic, anaerobic	carbon fixation using the reductive TCA cycle
Gammaproteobacteria	254	176	28	thermophilic, mesophilic, psychrophilic, psychrotolerant, aerobic, anaerobic, peizophilic, deep sea, halophilic, polar ice, soil, sediments, permafrost, 33 distinct sequences from species of *Psychrobacter,* 10 distinct sequences from species of *Halomonas* (halophilic), some produce intracellular gas vesicles, some are animal associated	nitrogen fixation, nitrate reduction, nitrite respiration, denitrification, sulfur oxidation, chemolithoautotrophs, iron oxidation, mineralization of aromatics, carbon fixation using the reductive pentose phosphate cycle
Uncultured Proteobacteria	8	5	2	unknown	unknown
**Spirochaetes**	3	3	0	animal pathogens	heterotrophic
**Tenericutes**	4	4	0	saprobes and arthropod pathogens/symbionts, anaerobic	heterotrophic
**Verrucomicrobia**	3	1	0	freshwater, soil, symbionts of protists and nematodes, aerobic	heterotrophic
**Uncultured Bacteria**	1839	1492	278	Sequences similar to those from uncultured and unidentified species, many from other environmental metagenomic studies	Unknown
**ARCHAEA**	2	0	0	deep hydrate-bearing sediment, peizotolerant, psychrotolerant	Methanotrophic, carbon fixation using the reductive acetyl-CoA pathway
**EUKARYA**	**221**	**124**	**27**		
**Amoebozoa**	1	1	0	*Nolandella* sp.; aquatic; feed on bacteria, diatoms, nematodes, fungi, protozoans and organic matter	Heterotrophic
**Archaeplastida**	74	28	9		
Chlorophyta	10	5	4	Antarctic and polar green algal species	carbon fixation using the reductive pentose phosphate cycle
Rhodophyta	1	0	0	Antarctic red alga	carbon fixation using the reductive pentose phosphate cycle
Streptophyta	63	23	5	Pollen from lake sediments or from glacial deposition?	(carbon fixation using the reductive pentose phosphate cycle)—non-viable?
**Chromalveolata**	12	6	2	diatoms, heterokonts, predatory protists, dinoflagellates, ciliates, Antarctic, aquatic	carbon fixation using the reductive pentose phosphate cycle, heterotrophic
**Excavata**	2	0	0	freshwater species	heterotrophic
**Opisthokonta**	115	79	10		
Animalia	24	10	3		
Arthropoda	16	8	0	Arctic, Antarctic, aquatic. (e.g., *Daphnia* sp., Ellipura, Branchiopoda, Entomobryiadae).	heterotrophic
Bilateria	1	0	1	Deep sediment environmental sample	unknown
Chordata	3	1	0	Aves, from meteoric ice or contaminant?	heterotrophic
Cnideria	1	0	0	Small sea anemone, lives in soft sediment with water salinities of 9 to 52 ppt at temperatures from −1 to 28 °C.	heterotrophic
Mollusca	1	0	1	*Nutricola* sp., cold water marine bivalve that burrows into sediments.	heterotrophic
Rotifera	1	1	0	Survives under extreme conditions; feed on detritus, bacteria, algae and protists.	heterotrophic
Tardigrada	1	0	1	Hardy animal, eats rotifers and algae, can survive from approximately −270 to 150 °C	heterotrophic
Fungi	91	69	7		
Ascomycota	48	34	4	Antarctic, polar, aquatic, soil	heterotrophic
Basidiomycota	29	24	0	Antarctic, polar, psychrophilic, psychrotolerant	heterotrophic
Mucorales	1	0	1	Aquatic, parasitic on arthropods	heterotrophic
Uncultured fungi	13	11	2	unknown	unknown
**Rhizaria**	1	0	0	Freshwater, *Paulinella* sp.	heterotrophic
**Uncultured** **eukaryotes**	16	10	6	unknown	unknown

^a^ Sequences ≥200 nt were submitted to NCBI GenBank and were assigned accession numbers, while those shorter than 200 nt could not be submitted to NCBI GenBank, and therefore do not have accession numbers. Totals based on BLAST searches using pyrosequencing reads; ^b^ Ecological, physiological and other characters were based on information from the sequenced organisms identified in the BLAST searches. Sources of information were NCBI descriptions, publications cited in the NCBI descriptions and web sources (see Supplementary Tables S1–S6).

## 2. Results and Discussion

### 2.1. Summary of Results

Two samples, each consisting of meltwater from two accretion ice core sections, were analyzed for this research. One (termed “V5”) contained meltwater from two ice core sections (3,563 m and 3,585 m; Figure 1) that accreted in the vicinity of the shallow embayment on the southwestern corner of Lake Vostok. The other (termed “V6”) contained meltwater from two ice core sections (3,606 m and 3,621 m) that accreted on the western side of the southern basin. Sequences have been deposited in the NCBI (National Center for Biotechnology) GenBank database (accession numbers are provided in the Experimental Section; BLAST results are presented in Supplementary Tables S1–S10 [22].). For the V5 sample, 36,754,464 bp of sequence data was obtained that included 94,728 high quality 454 sequence reads, with mean lengths of 388 bp. For the V6 sample, 1,170,900 bp of sequence data was obtained that included 5,204 high quality reads, with mean lengths of 225 bp. The lower quantity of sequence data for V6 is consistent with our previous results from the same core sections indicating much lower concentrations of cells and viable cells in the V6 ice core sections compared to the V5 ice core sections [16,17]. However, the lower number of sequences and the shorter average read lengths also might indicate that the nucleic acids in this sample were degraded to a greater extent than those in the V5 sample. Overall, approximately 15% of the sequences were unique, while the remaining 85% were additional copies from the unique set of sequences. A total of 3,718 unique sequences were retrieved from V5 (3,146 were rRNA gene sequences), of which 1,863 could be classified to species (Table 1; Supplementary Tables S1–S6 [22]), and 184 unique gene sequences were retrieved from V6 (111 were rRNA gene sequences), of which 133 could be classified to species (Table 2; Supplementary Tables S7–S10). Approximately 94% of the unique sequences in V5 and 85% in V6 were from Bacteria. Sequences closest to those from species of autotrophs and heterotrophs were present. Only two unique Archaea sequences were found (both in V5), and they were most similar to species of methanotrophs from deep-ocean sediments. The remaining sequences were closest to those from species of Eukarya (6% in V5 and 15% in V6), including more than 200 unique sequences from multicellular organisms, most of which were Fungi (primarily ascomycetes and basidiomycetes). In general, the species indicated by the sequence comparisons were organisms specific to lakes, brackish water, oceans/seas, soil, lake sediments, deep-sea sediments, deep-sea thermal vents, animals and plants.

**Table 2:** Summary of sequence results for V6 (total of 133 classified taxonomically; or 184, including sequences that cannot be classified to species). The number of sequences in each taxon, ecology/physiology of each classified taxon and species characteristics are presented. Sequences are grouped by Domain and Phylum.

Taxon	Unique gene sequences	Unique rRNA gene sequences ^a^		Ecology and physiology ^b^	Species characteristics ^b^
		≥200 nt <200 nt			
**BACTERIA**	**155**	**69**	**21**		
**Actinobacteria**	14	1	4	fish pathogen, psychrophilic, ocean/lake sediments	chemoorganotrophic heterotrophic
**Bacterioidetes/Chlorobi**	1	0	0	psychrophilic, alkalaiphilic, aerobic	heterotrophic
**Chloroflexi**	1	0	1		
**Deinococcus/Thermus**	1	0	0	thermophilic, radiophilic, some associate with cyanobacteria	chemoorganotrophic heterotrophic
**Firmicutes**	16	5	0	alkalaiphilic, thermophilic, mesophilic, psychrophilic, soil/sediments, anaerobic, some parasitic/symbiotic on animals	heterotrophic, nitrate reduction
**Fusobacteria**	1	0	0	mesophilic, parasitic on animals, anaerobic	heterotrophic
**Proteobacteria**	71	27	6		
Alphaproteobacteria	8	5	0	mesophilic, psychrophilic, aerobic, acid tolerance, aquatic, sediments, animal symbionts	nitrogen fixation, heterotrophic, carbon fixation using the reductive pentose phosphate cycle
Betaproteobacteria	22	6	3	annelid symbiont, annelid associated, Arctic soils, aquatic, Antarctic marine, intracellular gas vacuoles, high amounts of 16:1 ω7c fatty acids, psychrophilic, mesophilic, thermophilic, aerobic, highly adaptable, hot springs, (e.g., *Thiobacillus* sp., related to *Hydrogenophilus thermoluteus*, previously reported by Bulat *et al*. 2004 [20,21] Lake Vostok accretion ice at 3,607 m depth)	nitrogen fixation, chemoorganotrophic heterotrophic, aromatic hydrocarbon degradation, nitrous oxide reduction, arsenic oxidation, arsenic reduction, inorganic sulfur oxidation, chemolithoautotroph,, hydrogen oxidation, carbon fixation using the reductive pentose phosphate cycle
Gammaproteobacteria	39	14	3	fish intestinal symbionts (2 species), nematode associated, animal associated, plant associated, aquatic, soil/sediment, thermophilic, mesophilic, psychrophilic, anaerobic, aerobic, halotolerant	nitrogen fixation, nitrate reduction, nitrite respiration, heterotrophic, carbon fixation using the reductive pentose phosphate cycle
Uncultured Proteobacteria	2	2	0		
**Uncultured Bacteria**	50	36	10	sequences similar to those from uncultured and unidentified species, many from other environmental metagenomic studies	unknown
**EUKARYA**	**29**	**12**	**9**		
**Archaeplastida**	2	1	1		
Streptophyta	2	1	1	pollen from lake sediments or from glacial deposition?	(carbon fixation using the reductive pentose phosphate cycle)—non-viable?
**Opisthokonta**	26	10	8		
Animalia	5	0	0		
Arthropoda	5	0	0	aquatic, Acari, parasitic	heterotrophic
Fungi	22	10	8		
Ascomycota	13	7	3	aquatic, one grows on marble and limestone, one isolated from mid-ocean hydrothermal vents, some from sediments, one can use methanol as a carbon source, Antarctic species	heterotrophic
Basidiomycota	4	0	4	Antarctic, marine, aquatic	heterotrophic
Uncultured fungi	4	3	1	unknown	unknown
**Uncultured eukaryote**	1	1	0	unknown	unknown

^a^ Sequences ≥200 nt were submitted to NCBI GenBank and were assigned accession numbers, while those shorter than 200 nt could not be submitted to NCBI GenBank, and therefore do not have accession numbers; ^b^ Ecological, physiological and other characters were based on information from the sequenced organisms identified in the BLAST searches. Sources of information were NCBI descriptions, publications cited in the NCBI descriptions and web sources (see Supplementary Tables S7–S10).

### 2.2. Extremophiles

A large number of the sequences were most similar to those from psychrophilic and psychrotolerant species (Table 1, Table 2; Supplementary Tables S1–S10). Within the Gammaproteobacteria, there were 33 unique sequences closest to various *Psychrobacter* species (most with rRNA SSU gene identities of greater than 97%), all described as psychrophiles. Also present were sequences closest to psychrophilic or psychrotolerant species of Actinobacteria, Alphaproteobacteria, Archaea, Archaeplastida, Bacteroidetes, Betaproteobacteria, Firmicutes, Chromalveolata, and Opisthokonta (both Animalia and Fungi). Conversely, there were many sequences that were closest to those from several thermophilic species (many with rRNA SSU gene identities greater than 97%). While most were found in V5 ice (46 with rRNA sequence identities greater than 97% to known taxa), a few were found in V6 ice (2 with rRNA sequence identities greater than 97% to known taxa). A number of sequences most similar to those of sulfur oxidizing bacteria were found in V5 (Table 1, Table 2). Previously, several gene sequences from a thermophilic bacterium, *Hydrogenophilus thermoluteolus* were reported from the Lake Vostok accretion ice [20,21].

**Figure 2:**
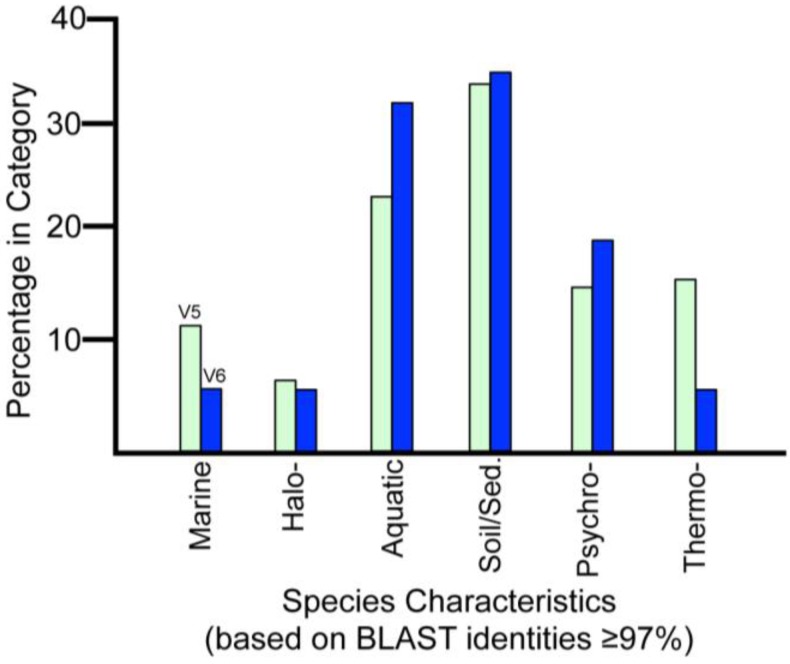
Comparisons of percentages of sequences according to characteristics of the closest species. Bars represent the percentages of sequences in V5 (green bars, N = 338) and V6 (blue bars, N = 38) that could be categorized with sequence identities ≥97% to NCBI sequences. Halo- = halophilic or halotolerant; Sed. = from lake or ocean sediments; Psychro- = psychrophilic or psychrotolerant; Thermo- = thermophilic or thermotolerant.

Several sequences from marine species, as well as from halophilic and halotolerant species, were present in the metagenome/metatranscriptome data set (Table 1, Table 2). Sequences closest to *Jeotgalicoccus halotolerans*, *Nesterenkonia halotolerans*, and other halophilic bacteria were found in V5 accretion ice, several of which have rRNA SSU gene identities greater than 98%. Some of these species were alkalaitolerant. A number of sequences in V5 and V6 were most similar to sequences from marine species, including marine bacteria (many with rRNA identities above 97%), a sea squirt-associated bacterium (99% rRNA SSU gene identity to *Pseudomonas xanthomarina*), an oyster pathogen (100% identity to a hypothetical protein from *Perkinseus maratimus*), a sea anemone (78% identity to a hypothetical protein from *Nematostella vectensis*, a small sea anemone, related to *Hydra* spp.) and a marine mollusk (100% rRNA SSU identity to *Nutricola tantilla*). The presence of marine, halophilic and halotolerant species is suggestive of marine layers, or other regions with high ion concentrations, within the lake or lake sediments. Saltwater layers and submarine brine lakes have been reported at the bottom of the Mediterranean Sea and Gulf of Mexico [25,26]. The number of V5 sample sequences with ≥97% identities to psychrophiles and thermophiles were roughly equal (46 and 49, respectively).The combination of possible halophiles, psychrophiles and thermophiles (at the ≥97% identity levels; Table 1, Table 2; Figure 2), in addition to higher concentrations of ions and particulate matter in the V5 sample, all are suggestive of a diversity of conditions in the southwestern region of the lake. In the V6 samples, there were more sequences (sequence identities ≥97%) closest to psychrophiles (7) than thermophiles (2). This is consistent with the presence of hydrothermal activity in the vicinity of the shallow embayment, and colder conditions in the main basin.

### 2.3. Metabolic Classification

We considered only sequences whose species and functional characteristics had been clearly identified. Genes for glycolysis, the TCA (tricarboxylic acid) cycle and genes for the synthesis of most amino acids were present (Supplementary Figures S1–S5). Incomplete pathways were found for the synthesis of arginine, histidine and proline. Alternate enzymes might be used for these processes, as determination of some sequences could not be made unambiguously. Some of the genes for each of these pathways were present, and therefore it is assumed that some of the unidentified sequences may encode for the missing enzymes in the pathways.

**Figure 3:**
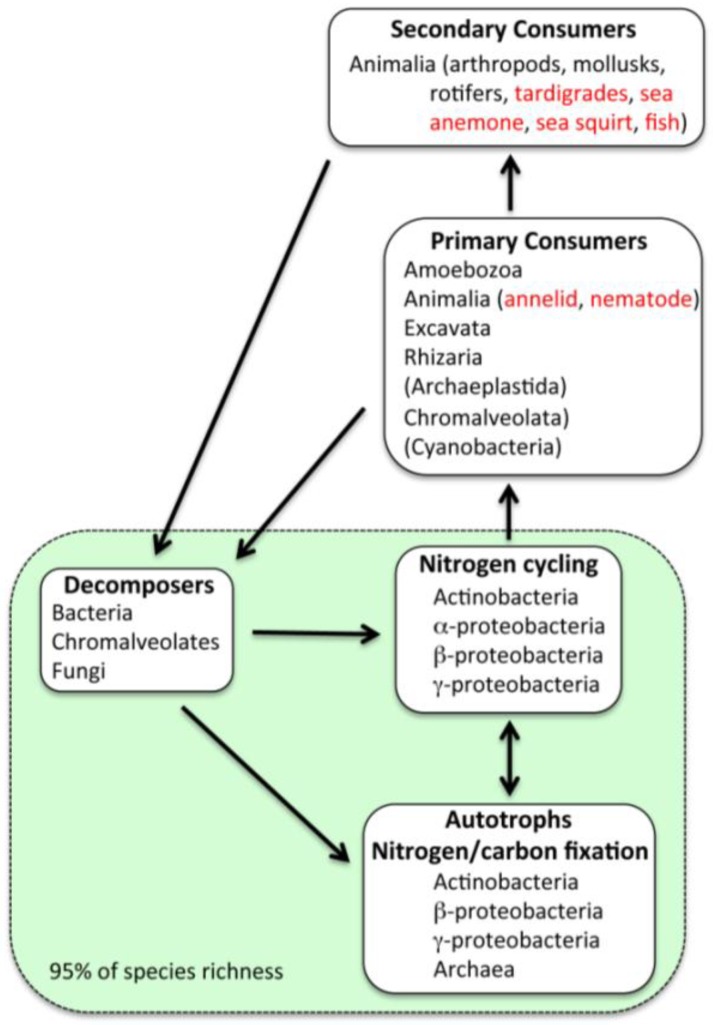
Ecological characterization of organisms based on BLAST search results from the V5 and V6 metagenomic/metatranscriptomic sequences. The taxonomic classifications listed are based on the highest identities to sequences from species within the taxa that have been documented to have the functions specified in the boxes. These have been grouped primarily by Phylum for simplicity. Greater than 95% of the taxa are either primary producers (autotrophs), bacteria involved in the nitrogen cycle, or decomposers, including bacteria and fungi, and a few chromalveolates (shaded green box). Primary and secondary consumers comprise less than 5% of the species richness (*i.e.*, number of unique sequences). Organisms listed in black font were determined by sequences that exhibited ≥97% sequence identity with sequences in GenBank (NCBI). Organisms listed in red font exhibited <97% sequence identity or were suggested because the sequences were closest to symbionts, parasites or pathogens of those organisms (at ≥97% sequence identity).

Sequences closest to those from bacteria capable of nitrogen fixation, nitrosification, nitrification, nitrate reduction, denitrification, anammox, assimilation and decomposition were present (Figure 3). Several types of nitrogen fixing bacteria were indicated by the sequences, including species of *Azospirillum*, *Azotobacter*, *Bacillus*, *Burkholderia*, Cyanobacteria, *Frankia*, *Klebsiella*, *Rhizobium*, *Rhodobacter*, *Rhodopseudomonas* and *Sinorhizobium*. Sequences of nitrifying bacteria included species of *Methylococcus*, *Nitrobacter*, *Nitrococcus*, *Notrosococcus* and *Nitrosomonas*. Species important in other parts of the nitrogen cycle were within the genera *Alkaligenes*, *Bacillus*, *Clostridium*, *Micrococcus*, *Paracoccus*, *Proteus*, *Pseudomonas*, *Streptomyces* and *Thiobacter*. Several sequences from planctomycetes were closest to sequences from species capable of anammox metabolic processes in marine environments.

Sequences of genes and organisms involved in many phases of carbon fixation cycles and pathways were found (Figure 3). Three forms of carbon fixation were indicated. The sequences indicated that most of the organisms utilize either the reductive TCA (rTCA) cycle (α-, δ- and ε-proteobacteria, and Chlorobi) (Table 1, Table 2) or the reductive pentose phosphate (rPP; Calvin-Benson) cycle (Archaeplastida, Chromalveolates, Cyanobacteria, and α-, β-, and γ-Proteobacteria) [27]. Based on the frequency of gene sequences, the most common mode of CO_2_-fixation was the rTCA cycle (Table 1, Table 2), while the rPP cycle was the second most common. The two Archaea in V5 may fix carbon via the reductive acetyl-CoA (rACA) pathway [27]. However, mRNA gene sequences for enzymes in this pathway were not found in searches of the metagenome/metatranscriptome data set.

A large number and diversity of sequences from phototrophs were present in the accretion ice, including 228 cyanobacterial, 11 algal, 12 chromalveolate, and other unique sequences. Sequences for many of the genes involved in the light reactions of photosynthesis in cyanobacteria were found in the accretion ice. These included light-independent photochlorophyllide reductase and oxidase, phycocyanobilin oxidoreductase, a phycoerythrin subunit and several genes involved in carotenoid biosynthesis. However, gene function was not measured in this study, and it is possible that some of the gene sequences were from pseudogenes, that they are inactive genes, or they originated from organisms once entrapped in the meteoric ice.

### 2.4. Eukaryotes

While only about 6% of the unique sequences were closest to eukaryotes (221 from V5, 87 of which have sequence identities of ≥97% with sequences on NCBI; and 29 from V6, 24 of which have identities ≥97%), diverse taxonomic groups were represented. Most of the sequences were most similar to those from Fungi (91 sequences in V5 and 22 in V6), including one rRNA SSU sequence that was 99% similar to a marine fungus sequence that previously had been recovered from a deep-sea thermal vent [28]. Several sequences from species of Animalia were found, including 21 sequences closest to those from arthropods (16 in V5 and 5 in V6), many of which are predatory or parasitic, including sequences closest to species of *Daphnia* (planctonic crustaceans; 98% identity), Ellipura (≥95% identity with species of springtails, some of which are aquatic or marine), Branchiopoda (fairy shrimp, primarily freshwater; 93% identity) and Entomobryidae (slender spingtails, some of which are aquatic; 89–98% identity). Additionally, V5 contained sequences closest to an unidentified bilaterian, a rotifer (closest to *Adineta* sp., which is a hardy, cosmopolitan, freshwater species; 98% identity), a tardigrade (closest to *Milnesium* sp., which is a hardy, predatory, cosmopolitan, freshwater species; 93% identity), a mollusk (*Nutricola tantilla*, a small [maximum diameter of 9 mm] marine bivalve that lives in sediments to about 120 m water depth; 100% identity) and a cniderian (related to *Nematostella* sp., a small sea anemone; 78% identity). Several Archaeplastida sequences were found in V5 and V6 (most with sequence identities of ≥99%).

Sequences closest to an uncultured lobster gut bacterium (98% identity), *Verminephorbacter* sp. (an annelid nephridia symbiont; 92% identity), *Renibacter salmonarium* (a salmonid fish pathogen; 98% identity), a rainbow trout intestinal bacterium T1 (93% identity) and *Photorhabdus asymbiotica* (a nematode symbiont; 98% identity) all were found in the V6 accretion ice sample. Additionally, sequences closest to *Carnobacterium mobile* (associated with fish; 100% identity), *Macrococcus* sp. (a bivalve-associated bacterium; 97% identity), *Pseudomonas xanthomarina* (associated with sea squirts; 99% identity) and *Mycobacterium marinum* (associated with fish; 99% identity) were found in the V5 accretion ice sample. All of the species are dependent on intimate associations (symbiotic or parasitic) with their eukaryotic hosts, which are crustaceans, annelids, tunicates, nematodes or fish. Species of all of these animal groups have been found in the vicinity of deep-sea thermal vents and elsewhere in marine and aquatic environments [29,30,31,32,33,34,35,36,37]. Additional indications of animals in the lake came from sequences of several species in the Enterobacteriaceae, which were present in both the V5 and V6 samples. These included sequences closest to several strains/species of *E. coli*, *Erwinia*, *Klebsiella*, *Salmonella*, and *Shigella* (most with sequence identities ≥97%), all of which are found in the digestive systems of fish and other aquatic and marine animals. In addition, sequences closest to those from species of Fusobacteria (some with ≥97% identities) that are parasitic on animals, Alphaproteobacteria (some with ≥97% identities) that are animal symbionts, and Tenericutes that are arthropod symbionts and pathogens (up to 91% sequence identity) were found in the V5 sample. 

In V5, there were 16 sequences closest to those from single-celled eukaryotic species. These included sequences closest to species of Excavata (≥98% identity), Rhizaria (closest to *Paulinella* sp., a freshwater phototroph; 94% identity), Amoebozoa (*Naeglaria gruberei*, 98% identity; *Nolandella* sp., marine, 88% identity) and Chromalveolata (10 unique sequences, including two Ciliophora, 99% identity each; three bacillariophytes, *Stephanodiscus* sp.—99%, *Stephanodiscus* sp.—100%, *Hatzschia* sp.—95% identity; three heterokonts, *Aphanomyces euteiches*—98% identity, *Botrydiopsis constricta*—99% identity, *Halosiphon tomentosus*—97% identity); a cryptophyte, *Cryptomonas paramecium*—100% identity; and a member of Perkinsea, obligate parasite of mollusks—100% identity. While some of these eukaryotes are free-living, many are symbionts, parasites or commensals on multicellular organisms, including animals and plants. Several multicellular organisms were indicated by the sequence data, but the sequences closest to symbionts and parasites suggest that a more diverse set of multicellular eukaryotes might exist in the lake.

### 2.5. Possible Marine Environment in Lake Vostok

**Figure 4:**
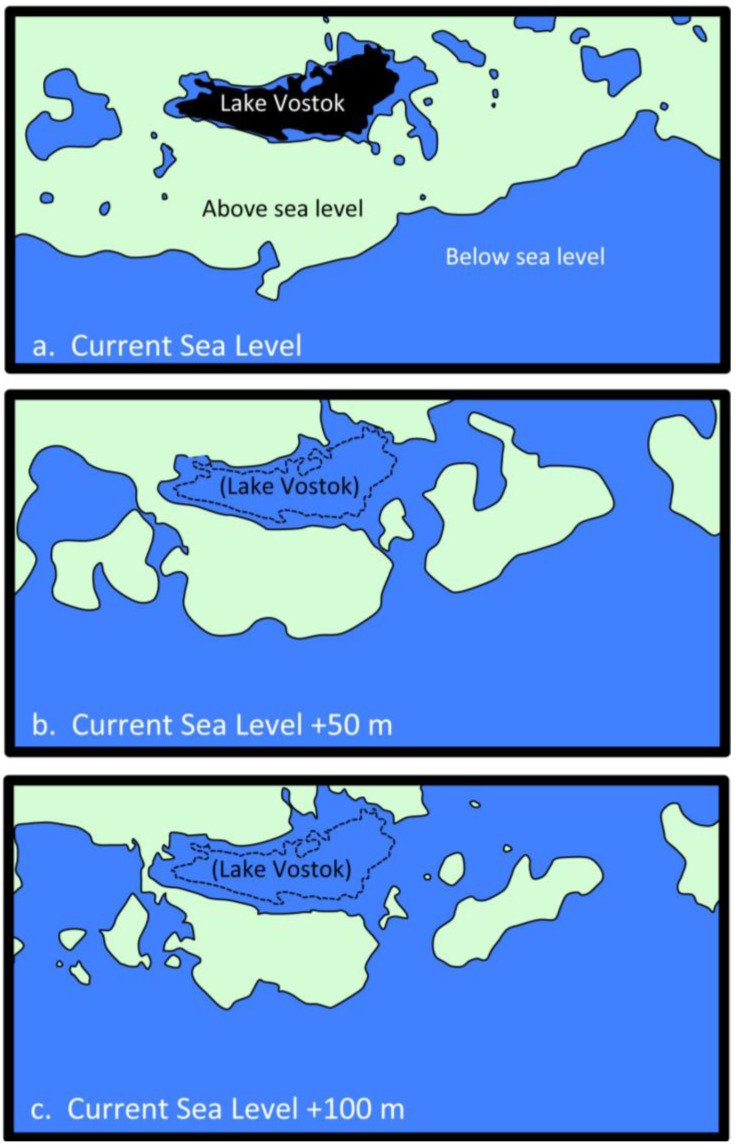
Sea level changes relative to the current level of water in Lake Vostok (based on reference [24]). (**a**) Current mean sea level. Blue indicates areas below sea level if all of the ice was removed. Light green indicates areas above sea level. The upper surface of Lake Vostok (black region) is approximately 200 m below current sea level; (**b**) View of the same region with a 50 m sea level rise. Current outline of Lake Vostok is indicated by the dashed line; (**c**) View of the same region with a 100 m sea level rise.

Organisms in Lake Vostok have had millions of years to adapt and evolve. The lake has been continuously ice-covered for approximately 15 million years [1,4,10,23,24,38]. During parts of the Miocene (15 to 25 million years ago, mya), it was intermittently ice-free, and previous to that, a cooling period from 25–34 mya led to the lake to be ice-covered much of the time [38]. However, prior to 35 mya (during the Eocene), most of Antarctica (including the Lake Vostok region) was free of ice, sea levels were higher and extensive complex ecosystems were present on the continent, complete with lakes and streams, as well as diverse sets of microbes, plants (including extensive forests), fungi and animals. During these times, Lake Vostok probably contained a species-rich biota. Currently, Lake Vostok is below sea level. Currently, it is separated from regions that once were occupied by ocean water by low ridges that are approximately 20–50 m above current mean sea level [23,24,38]. However, 35 million years ago, sea levels were between 50–100 m higher. Therefore, this low area may have been a straight that connected Lake Vostok with the ocean, thus making it a large marine bay (Figure 4). Our metagenomic/metatranscriptomic data set included sequences that are most similar to sequences from marine organisms, including many Bacteria, halophiles, a marine mollusk, a sea anemone, a marine thermal vent fungus and two Archaea that previously were described from deep-ocean sediments. As temperatures in Antarctica began to cool and sea levels dropped about 34 million years ago, Lake Vostok become ice covered and isolated from the ocean [24]. Aerobic organisms that survive in cold and partially shaded aquatic environments could have survived in the epilimnion. Anaerobes would have been limited to the benthic and sediment regions. Hydrothermal regions may have been present much of the time, because rifting of the region began more than 60 million years ago [23]. As freshwater from the melting glacier flowed into the lake, ion gradients were likely to develop. These gradients are common in other ice-covered Antarctic lakes [39,40,41,42], and the differences in ion concentrations in the embayment compared to the main basin are consistent with this hypothesis. Hydrothermal activity in the vicinity of the embayment might cause mixing of any stratified layers in the lake in that region, leading to the higher ion and mineral inclusion concentrations in the accretion ice from that region.

Once current continuous ice-cover began approximately 15 million years ago, additional selection would have occurred as light intensities decreased, and oxygen concentrations and water pressures increased. Although extinctions would probably have been extensive, it is unlikely that all life would disappear from the lake and its sediments. Based on the number of unique species matches in BLAST searches of our accretion ice metagenomic/metatranscriptomic data set, we estimate the total number of species to be at least 1,996 in Lake Vostok, of which over 90% (approximately 1,800) are Bacteria. This converts to approximately 4 species mL^−1^, assuming that the concentration of nucleic acids in the accretion ice is representative of the concentration of nucleic acids and organisms in the lake water, and that the samples are representative of the lake in general. However, as ice forms, it can force out many components, including some molecules, minerals and cells. Therefore, the concentrations of organisms and nucleic acids are probably higher in the lake water than in the accretion ice. The calculated concentration is below the number of species found in ocean water, sediments and most freshwater lakes [43,44,45,46]. Because the accretion ice represents primarily water at the surface of the lake, species density and richness in the lake could be much higher in some locales. In any case, a relatively high number of sequences matching sequences from a diverse set of species were found in Lake Vostok accretion ice. The 15–35 million years of coverage by ice appears to have been ample time for many organisms to adapt to the extreme conditions that developed in the lake, which has likely led to numerous speciation events.

## 3. Experimental Section

### 3.1. Acquisition and Processing of Ice Core Sections

All ice core sections were selected from the USGS NICL (United States Geological Survey, National Ice Core Laboratory, Denver, CO, USA). They were selected based on desired depths and for the absence of cracks (to avoid possible external contamination). They were shipped frozen to our laboratory. Sections were surface sterilized according to a tested method that assures destruction and removal of all surface contaminating cells and nucleic acids, while preserving cells and nucleic acids frozen in the ice [16,17,47,48]. Briefly, quartered ice core sections, 6–16 cm in length (total volume approximately 125 mL), were warmed at 4 °C for 30 min (to avoid thermal shock and cracking) before surface decontamination. The work surfaces in a room (under positive pressure) separate from the main laboratory were treated with 0.5% sodium hypochlorite, 70% ethanol and UV irradiation for one hour prior to surface sterilizing and melting the ice core sections. Inside a sterile laminar flow hood, the ice core sections were surface decontaminated by total immersion in a 5.25% sodium hypochlorite solution (pre-chilled to 4 °C for at least 2 h) for 10 s followed by three rinses with 800 mL of sterile water (4 °C, 18.2 MΩ, <1 ppb [parts per billion] total organic carbon, TOC, and autoclaved). Then, the core section was transferred into a sterile funnel and melted at room temperature by collection of 25–50 mL aliquots. This protocol significantly reduces the risk of contamination of the ice core meltwater samples [47,48]. The meltwater was then frozen at −20 °C. Sample V5 included meltwater from Vostok 5G core sections at depths of 3,563 and 3,585 m, corresponding to type 1 ice that accreted in the vicinity of the embayment. Sample V6 included core sections 3,606 and 3,621 m, corresponding to type 2 ice that accreted over a portion of the southern main basin of Lake Vostok (Figure 1). A total of 250 mL of meltwater was used for each sample (approximately 125 mL from each ice core section). The meltwater samples were filtered sequentially through 1.2, 0.45 and 0.22 μm Durapore filters (Millipore, Billerica, MA, USA). The filters were stored at −80 °C for future reference. Then, the filtered meltwater was subjected to ultracentrifugation at 100,000 xg for 16 h to pellet cells and nucleic acids. Two control samples (purified water, 18.2 MΩ, <1 ppb TOC; and the same water, autoclaved and subjected to concentration by ultracentrifugation) also were processed using the same protocols. The V5, V6, and control samples were ultracentrifuged on different days to lessen potential cross-contamination. Pellets were rehydrated in 50 µL 0.1× TE (1 mM Tris [pH 7.5], 0.1 mM EDTA).

### 3.2. DNA and RNA Extraction

Nucleic acid extraction was performed using MinElute Virus Spin Kits (QIAGEN, Valencia, CA) and eluted in 150 µL AVE buffer (water with 0.04% sodium azide). This kit isolates both RNA and DNA. The eluted nucleic acids were further concentrated by precipitating overnight at −20 °C with 0.5 M NaCl in 80% ethanol. They were then pelleted by centrifugation at 16,000 × *g* for 15 min, washed with cold 80% ethanol and centrifuged at 16,000 × *g* for 5 min. They were dried under vacuum, and then they were resuspended in 15 µL 0.1× TE.

### 3.3. cDNA Synthesis and Amplification of cDNA and DNA

Complementary DNAs (cDNAs) were synthesized from the extracted RNAs. The procedure was performed using a SuperScript Choice cDNA kit (Invitrogen, Grand Island, NY, USA), according to the manufacturer’s instructions, using 10 µL of the extracted RNA and 80 pmol of random hexamer primers. The cDNA was then mixed with 10 µL of extracted DNA (less than 1 ng/µL) from the same meltwater sample, and *Eco*RI (Not I) adapters (AATTCGCGGCCGCGTCGAC, dsDNA) were added using T4 DNA ligase. The final concentration of components in each reaction for addition of *Eco*RI adapters was: 66 mM Tris-HCl (pH 7.6), 10 mM MgCl_2_, 1 mM ATP, 14 mM DTT, 100 pmols *Eco*RI (*Not* I) adapters and 0.5 units of T4 DNA ligase, in 50 µL total volume. The reaction was incubated at 15 °C for 20 h. Then, the reaction was heated to 70 °C for 10 min to inactive the ligase. [Note: The cDNAs and DNAs were mixed in order to maximize the biomass of nucleic acids, necessary for successful pyrosequencing. Thus, the cDNA comprised the metatranscriptomic fraction (of which most was from rRNA) and the DNA comprised the metagenomic fraction of each sample].

The products were size fractionated by column chromatography. Each 2 mL column contained 1 mL of Sephacryl^®^ S-500 HR resin. TEN buffer (10 mM Tris-HCl [pH 7.5], 0.1 mM EDTA, 25 mM NaCl; autoclaved) was utilized to wash the columns and elute the samples through the columns. Fractions of approximately 40 µL were collected by chromatography. After measurement of the volume of each fraction, fractions 6–18 were precipitated to concentrate the DNA. Concentration was accomplished by adding 0.5 volumes (of the fraction size) of 1 M NaCl, and two volumes of −20 °C absolute ethanol. After gentle mixing, each was left to precipitate at −20 °C overnight. The fractions were centrifuged in a microfuge for 20 min at room temperature and decanted. Then, the pellets were washed with 0.5 mL of −20 °C 80% ethanol and centrifuged for 5 min. Finally each of the DNA pellets was dried under vacuum and rehydrated in 20 µL of 0.1× TE buffer. 

After resuspension, fractions 6–18 were subjected to PCR amplification using *Eco*RI (*Not*I) adapter primers (AATTCGCGGCCGCGCTCGAC). The samples were amplified using a GeneAmp PCR Reagent Kit (Applied Biosystems, Carlsbad, CA, USA). Each reaction mixture contained: 10 mM Tris-HCl [pH 8.3], 50 mM KCl, 1.5 mM MgCl_2_, 0.001% (w/v) gelatin), 5 pmol each dNTP (dATP, dCTP, dGTP, and dTTP), 1 U Ampli*Taq* DNA polymerase and 50 pmols *Eco*RI (*Not*I) adapter primers (AATTCGCGGCCGCGCTCGAC), each in 25 µL total volume. The thermal cycling program was: 94 °C for 4 min; then 40 cycles of 94 °C for 1 min, 55 °C for 2 min, 72 °C for 2 min; followed by an incubation for 10 min at 72 °C. A 1 µL aliquot of each was subjected to 1% agarose gel electrophoresis at 5 V/cm in TBE (89 mM tris, 89 mM borate, 2 mM EDTA [pH 8.0]), containing 0.5 µg/mL ethidium bromide, and visualized by UV irradiation. Fractions that excluded small (<200 bp) and large (>2.0 kb) fragments were used for further processing. Then, amplified products for each fraction of the desired size range (as above) were pooled (approximately 350 µL per sample), and were precipitated with NaCl and ethanol, washed, and dried (as above). Each was rehydrated in 35 µL of 0.1× TE.

### 3.4. Addition of 454 A and B Sequences by PCR Amplification

Each of the amplified samples was then reamplified using primers that contained *Eco*RI/*Not*I sequences on their 3' ends and 454-specific primers on their 5' ends (one primer with sequence A; underlined: CGTATCGCCTCCCTCGCGCCATCAGAATTCGCGGCCGCGTCGAC; and the other with sequence B; CTATGCGCCTTGCCAGCCCGCTCAGAATTCGCGGCCGCGTCGAC). The thermal cycling program was: 94 °C for 4 min; then 40 cycles of 94 °C for 1 min, 55 °C for 3 min, 72 °C for 3 min; followed by an incubation for 10 min at 72 °C. All PCR products were cleaned with a PCR purification kit (QIAGEN, Valencia, CA, USA). The amplicons were quantified on agarose gels (as above) to calculate concentrations (based on comparisons to plasmid pGEM4Z (Promega, Madison, WI, USA) standards on the same gel). After adjusting concentrations to approximately 1 µg/µL, 20 µg of each was sent to Roche 454 Life Sciences 454 Technologies (Roche, Branford, CT, USA) for 454 pyrosequencing using a 454 GS Junior System.

### 3.5. Sequence Analysis

The sequences were extracted from the data file and organized using Python (Python Software Foundation) on the Ohio Super Computer (OSC, Columbus, OH, USA). The sequences were deposited in the GenBank nucleotide database at the National Center for Biotechnology Information (NCBI; accession numbers: JQ997163–JQ997235; JQ997237–JQ997322; JQ997324–JQ997402; JQ997404–JQ997547; JQ997549–JQ998298; JQ998300–JQ998745; JQ998747–JQ999505; JQ999568–JQ999624; JQ999909–JQ999910; JQ997196–JQ997198; JQ997274; JQ997284; JQ997285; JQ997287; JQ997308; JQ997309; JQ997361; JQ997374; JQ997375; JQ997378; JQ997384; JQ997393; JQ997394; JQ997443; JQ997448; JQ997457; JQ997460; JQ997469; JQ997487–JQ997497; JQ997541; JQ997613; JQ997623; JQ997624; JQ997638; JQ997639; JQ997651; JQ997695; JQ997698; JQ997801; JQ997804; JQ997847; JQ998421; JQ998746; JQ999303; JQ999327; JQ999330; JQ999348; JQ999360; JQ999361; JJQ999365–JQ999369; JQ999371; JQ999492; JQ999493; JQ999635–JQ999829; JQ999837–JQ999897; JQ999899; JQ999901–JQ999905; JQ999507–JQ999509; JQ999512; JQ999515; JQ999518–JQ999521; JQ999523–JQ999526; JQ999529–JQ999530; JQ999533; JQ999538; JQ999540; JQ999545; JQ999549; JQ999552; JQ999554; JQ999556–JQ999564; JQ999567; JQ999629; JQ999631; JQ999830; JQ999831; JQ999833–JQ999835). They were assembled using MIRA 3.0.5 (Whole Genome Shotgun and EST Sequence Assembler [49]), using the following command line: job=denovo, genome, accurate,454. From the assembly, the average lengths for V5 and V6 were 539 and 318, respectively. The average quality scores were 39.7 and 40.2, respectively; maximum coverages were 3.3 and 6.2, respectively; and average coverages were 2.2 and 3.5, respectively. Initial taxonomic analyses were performed on MG-RAST [50] and Galaxy [51], Batch Mega-BLAST searches were performed to determine taxonomic and gene identities. The BLAST execution file was set up to retrieve the top 10 similar sequences, with e-value cutoffs of 10^−10^. They were subjected to batch BLASTN similarity searches on the OSC, and then sorted according to gene, taxon, and similarity e-values using FileMaker (FileMaker, Inc., Santa Clara, CA, USA). The top BLASTN hit was used to determine taxonomic classification (when genus and species names were provided), also considering the lengths and percent similarities of the matches. The sequences were divided into four category files: V5 rRNA genes, V6 rRNA genes, V5 mRNA genes and V6 mRNA genes (Full list of sequences and descriptions in reference [22], and presented in Supplementary Tables S1–S10. The rRNA gene results were used primarily to determine taxonomic classifications. Each species was then categorized according to temperature requirements, growth requirements, metabolic functions and ecological niche, based on NCBI descriptions, publications cited in the NCBI descriptions, and internet sources. Some mRNA sequences were used to determine or confirm species identifications, where possible.

### 3.6. Metabolic Analysis

Sequences were uploaded onto the KAAS-KEGG (KEGG Automatic Annotation Server; KEGG—Kyoto Encyclopedia of Genes and Genomes, Kyoto, Japan) pathway website and blasted against the default set of bacterial and eukaryotic genes [52]. The sequences were compared to known sequences from 40 taxa (23 provided on the KAAS-KEGG site, and 17 additional taxa added manually) The additional taxa (with abbreviations used for searches) were: *Cryptococcus neoformans* JEC21; *Thalassiosira pseudonana*; *Dictyostelium discoideum*; *Burkholderia mallei* ATCC 23344; *Campylobacter jejuni* NCTC11168; *Desulfovibrio vulgaris* DP4; *Caulobacter crescentus* CB15; *Micrococcus luteus*; *Acidobacterium capsulatum*; *Flavobacterium johnsoniae*; *Fibrobacter succinogenes*; *Fusobacterium nucleatum*; *Opitutus terrae*; *Gemmatimonas aurantiaca*; *Rhodopirellula baltica*; *Chlorobium limicola*; *Chloroflexus aurantiacus*. Based on the results from the KAAS-KEGG analysis, metabolic pathways that were present in our dataset were identified. Tables of enzymes that matched our sequences from each pathway were retrieved. Subsets of these are presented in Supplementary Figures S1–S5.

## 4. Conclusions

Lake Vostok accretion ice contains nucleic acids from a diversity of species. While many were closest to those from psychrophilic species, a large number of sequences were closest to those from thermophilic species. Sequences from both anaerobes and aerobes were represented, as well as halophiles, aquatic and marine species. The list of taxa included approximately 94% Bacteria and 6% Eukarya, including over 100 species of multicellular Eukarya. While most were fungi (primarily ascomycetes and basidiomycetes), a number of animals also were indicated, including a rotifer, tardigrade, nematode, bivalves, sea anemone, crustaceans, and possibly fish (as suggested by the presence of sequences that were most similar to those from bacterial symbionts and pathogens of fish species). The species indicated by the sequences include those that participate in many parts of the nitrogen cycle, as well as those that fix, utilize and recycle carbon. Because of the higher concentrations of nucleic acids and viable organisms in accretion ice compared to the overriding meteoric ice [16,17], it is likely that the organisms and nucleic acids in the accretion ice originated in the lake water. The indications of large numbers of marine, halophilic and halotolerant organisms suggest that marine layers, or other saline regions, might exist in the lake. They may have originated millions of years ago at a time when the lake might have been physically connected with the surrounding ocean. Therefore, Lake Vostok might contain a complex interdependent set of organisms, zones and habitats that have developed over the tens of millions of years of its existence.

## Supplementary Files

Supplementary File 1 Supplementary Materials (RAR, 2133 KB)
